# Supplementary material for: Imaging the Transverse Component of Optical Near-Fields in Resonant Photonic Structures
Source: ACS Photonics. 2026 Jul 6;13(14):4029–36. doi: 10.1021/acsphotonics.6c00984 (PMC13377604; doi:10.1021/acsphotonics.6c00984)
Supplement: Supplementary file 1 [file ph6c00984_si_001.pdf]

Imaging the transverse component of optical near-fields in resonant photonic structures

Petr Koutenský[1,*], Neli Laštovičková Streshkova[1], Stefanie Kraus[2], Peter Hommelhoff[2,3], and Martin Kozák[1,†]

[1]Department of Chemical Physics and Optics, Faculty of Mathematics and Physics, Charles University, Ke Karlovu 3, Prague 2, CZ-12116, Czech Republic.
[2]Physics Department, Friedrich Alexander University Erlangen-Nürnberg, Staudtstr. 7/B2, Erlangen, 91058, Germany
[3]Faculty of Physics, Ludwig Maximilian University Munich, Schellingstr. 4, Munich, 80799, Germany

* Email: petr.koutensky@matfyz.cuni.cz
† Email: m.kozak@matfyz.cuni.cz

7 pages, 3 figures, 0 tables

Experimental setup

The experimental setup is shown schematically in Figure 1.a. The electron-light interaction is studied in a scanning electron microscope Verios 5 UC (Thermo Fisher Scientific), which is modified for ultrafast operation. The electrons are photoemitted from the Schottky-type source using femtosecond laser pulses at the wavelength of 515 nm generated using second harmonic generation of the output of a Ytterbium-60 HE (AFS) femtosecond laser system with central wavelength of 1030 nm, pulse duration of 250 fs and repetition rate of 500 kHz. The laser pumps an optical parametric amplifier (OPA), which is used to generate infrared pulses with the central wavelength of 1.93 μm and FWHM duration of 110 fs, which are used to excite the optical near-fields of the resonant naosotructures. The experiments are performed with an electron kinetic energy of 28.6 keV. At this energy, the group velocity of the electrons is matched to the phase velocity of the first spatial harmonic of the optical nearfield mode of the periodic nanostructure. To allow sufficient transverse momentum resolution, we use the microscope setting that generates a beam with a low semi-divergence angle of 1 mrad using the highest current settings and introducing an objective aperture with diameter of 64 μm. Each electron pulse in the final focused low-divergence beam contains approximately 0.0085 electrons on average, as calculated from the laser repetition rate, exposure time, and number of detected electrons. The duration of the electron pulse was 800 fs (FWHM). The working distance is set to 15 mm.

The spatial resolution of the U4DSTEM imaging was characterized using contrast in the ultrafast STEM image. We used the line cut of the edge of the nanostructure which was fitted by error function. A standard edge resolution definition in electron microscopy (the distance of two points between which the contrast value drops from 65% to 35%) is applied to the fit to retrieve a value of 21 ± 1 nm. Figure S1 shows STEM data from Figure 2.b,d. The error is the maximum value spread for different edge contrast profiles in the same data.

The time delay between the electron pulse and the optical fields in the sample plane is controlled by using an optical delay line. The intensity of excitation is controlled by a combination of a half-wave plate and a polarizer for both the fundamental and the photoemission beams. The electron detector (hybrid pixel detector Timepix3, Advascope) is placed at a distance of $L = 16.4$ cm downstream of the sample plane. Acquisition of detector data is synchronized with the position of the electron beam in the sample plane, which is controlled externally using a PCIe-6323 card from National Instruments.

In general, the electron beam moves on the detector during STEM type of measurements unless it is compensated for. In our experimental setup with the scanned area depicted in Figure 2, the shift of the center of mass is negligible. Moreover, the amplitude of the transverse momentum change of the electrons are obtained by calculating a standard deviation of the electron distribution on the detector, which is independent of a shift of the center of mass.

Analytical formula for transverse momentum change

In this section, we describe the relation between the transverse momentum change of the electrons, which is plotted in Figs. 2 and 3, and the image formed by the scattered electrons on the detector. In our analysis, we take into account the finite spot size of the electron beam as well as the finite electron and laser pulse durations.

To calculate the scattering pattern of the electrons on the detector, we assume that the electron beam can be described by an initial density in the transverse momentum space, which accounts for the angular divergence of the electron beam. The transverse momentum distribution of electrons without interaction can be described in cylindrical coordinates using a step function:

$$h(p_r) = \begin{cases} 1, & p_r \leqq p_e, \\ 0, & p_r > p_e. \end{cases} \tag{1}$$

Here $p_r = \sqrt{p_x^2 + p_y^2}$ is the radial momentum component of the electron and we assume that the momentum component along the azimuthal angle $\varphi = \arctan(p_y/p_x)$ is zero $p_\varphi = 0$. When the electron distribution interacts with the optical near-field, the transverse momentum is modulated, leading to a scattering pattern

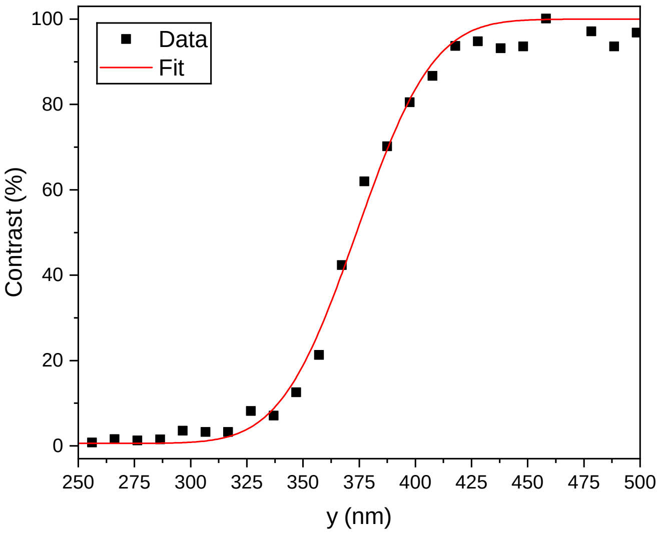

Figure S1: **Characterization of spatial resolution of U4DSTEM from the edge contrast.** Scatter plot shows normalized ultrafast STEM image intensity of the data in Figure 2b,d along a vertical slice though the center of the right pillar. To suppress the noise, the data is averaged from three horizontally adjacent pixels. Red line is an error function fit. The resolution of $21 \pm 1\,\mathrm{nm}$ is obtained as a distance of two points between which the contrast value drops from 65% to 35%. The error is the maximum value spread for different edge contrast profiles in the same data.

on the detector. The change of the electron transverse momentum $\Delta\mathbf{p}_\perp$ (1) is given by the integral of the transverse components of the Lorentz force $\mathbf{F}_\perp$ of the near-field acting on the electron along it's trajectory in the vicinity of the near field $\Delta\mathbf{p}_\perp = \int \mathbf{F}_\perp \,\mathrm{d}t$. Because the electron momentum does not change significantly during the interaction ($|\Delta\mathbf{p}| \ll |\mathbf{p}_0|$), we can apply the nonrecoil approximation. Practically, it means that the integral (1) representing the interaction inducing a momentum change is evaluated over the original classical trajectory of the electrons in the z direction. The momentum change for an electron traversing the near field in time Δt and with initial phase of the near field φ_0 is

$$\Delta\mathbf{p}_\perp = \Re \int_{-\infty}^{\infty} e\left[\tilde{\mathbf{E}}(\mathbf{r},\omega) + \mathbf{v}\times\tilde{\mathbf{B}}(\mathbf{r},\omega)\right]_\perp g(t-\Delta t)e^{-i\omega(t-\Delta t)+i\varphi_0}\mathrm{d}t, \tag{2}$$

where $g(t-\Delta t)$ is the laser pulse envelope normalized to 1 at maximum, with the characteristic time duration τ_l. The non-relativistic electron trajectory is related to the time of the interaction as $z(t) = vt$. Using the definition of the Lorentz force $\tilde{\mathbf{F}}_\perp = e\left[\tilde{\mathbf{E}} + \mathbf{v}\times\tilde{\mathbf{B}}\right]_\perp$ and assuming oscillations in z, modulated by a slow envelope, we write $\tilde{\mathbf{F}}_\perp(x,y,z,\omega) = \mathbf{F}_0(x,y,z)e^{ikz}$. With the substitution $u = t-\Delta t$, $z = v(u+\Delta t)$ the integral becomes

$$\Delta\mathbf{p}_\perp(\Delta t) = \Re\left[e^{i(\varphi_0+kv\Delta t)}\int_{-\infty}^{\infty}\mathbf{F}_0(x,y,v(u+\Delta t))g(u)e^{-i(\omega-kv)u}\,du\right]. \tag{3}$$

The spatial distribution of $\tilde{\mathbf{E}}(\mathbf{r},\omega)$, $\tilde{\mathbf{B}}(\mathbf{r},\omega)$ is confined to a spatial region $z_{\mathrm{int}} = 25\,\mu\mathrm{m}$, corresponding to an interaction time $\tau_{\mathrm{int}} = z_{\mathrm{int}}/v \approx 260\mathrm{fs}$ while the laser pulse envelope duration is $\tau_l = 110\,\mathrm{fs}$. In the approximation $\tau_l \ll \tau_{\mathrm{int}}$, which is still reasonable for this case, the spatial envelope of the near-field varies slowly during the presence of the laser pulse and we can approximate $\mathbf{F}_0(x,y,v(u+\Delta t)) \approx \mathbf{F}_0(x,y,v\Delta t)$. In an opposite limit situation, where $\tau_l \gg \tau_{\mathrm{int}}$, which is the case of a near-field localized around a nano-tip for example, the approach is a little different, approximating $g(t-\Delta t) \approx g(\Delta t)$, and leaving $\mathbf{F}(x,y,t)$ under

the integral[8]. Otherwise it leads similar results. In the case $\tau_{\text{int}} \approx \tau_l$, the integral in Eq. (3) needs to be computed explicitly.

In the case $\tau_l \gg \tau_{\text{int}}$ the total exchanged momentum is

$$\Delta\mathbf{p}_\perp = \Re\Big[\mathbf{F}_0(x,y,v\Delta t)e^{+ikv\Delta t+i\varphi_0}\tau_l\int_{-\infty}^{\infty}\frac{1}{\tau_l}g(u)e^{-i(\omega-kv)u}\,\mathrm{d}u\Big], \tag{4}$$

where the remaining integral $I_\omega(vk) = \int_{-\infty}^{\infty}\tau_l^{-1}g(u)e^{-i(\omega-kv)u}\,\mathrm{d}u$ is the velocity-matching condition. Without loss of generality, we assume perfect velocity-matching $\omega = kv$. We define the maximal possible exchanged momentum $\Delta\mathbf{p}_{\max} \equiv \max_{\Delta t}\mathbf{F}_0(x,y,v\Delta t)\tau_l I_\omega(kv)$, which is a vector with complex amplitude in general. We assume that the Lorentz force components F_x and F_y are in phase, and additional constant field phase is included in φ_0, then $\Delta\mathbf{p}_{\max}$ is a real vector. Lastly, we assume that we can approximate the Lorentz force spatial envelope by $\eta(\Delta t)$ such that $\mathbf{F}_0(x,y,v\Delta t) \approx \max_{\Delta t}\mathbf{F}_0(x,y,v\Delta t)\eta(\Delta t)$ We rewrite

$$\Delta\mathbf{p}_\perp = \Delta\mathbf{p}_{\max}\eta(\Delta t)\cos(\omega\Delta t + \varphi_0). \tag{5}$$

From there we easily find the density of electrons in the transverse momentum area $(p_x, p_x + dp_x) \times (p_y, p_y + dp_y)$

$$\rho(p_x,p_y) = \frac{\mathcal{N}}{2\pi\tau_e\Delta S N}\iint n(\Delta t)\delta^{(2)}[\Delta\mathbf{p}_\perp - \Delta\mathbf{p}_{\max}\eta(\Delta t)\cos(\omega\Delta t+\varphi_0)]\,\mathrm{d}\varphi_0\mathrm{d}\Delta t, \tag{6}$$

where $\delta^{(2)}$ is the 2D Dirac delta function, the integral is normalized to the angular period, electron pulse duration τ_e, the spot area in the momentum space ΔS in $(\mathrm{kg}\cdot\mathrm{m}\cdot\mathrm{s}^{-1})^2$ and the average number of electrons per unit time $\bar{n}$. An additional scaling constant $\mathcal{N}$ accounts for the normalization of the unit-less functions and will be determined in the end. The envelope $n(\Delta t)$ is the number electrons arriving at Δt per time unit. It is obvious, that the deflection will occur in the direction of $\Delta\mathbf{p}_{\max}$ and no spread will be present in the perpendicular direction. Without loss of generality, we assume that $\Delta\mathbf{p}_{\max} = (\Delta p_{\max}, 0)$ is along x and that $\Delta\mathbf{p}_\perp = (p_x, p_y)$

$$\rho(p_x,p_y) = \frac{\mathcal{N}}{2\pi\tau_e\Delta S\bar{n}}\iint n(\Delta t)\delta(p_y)\delta[\Delta p_x - \Delta p_{\max}\eta(\Delta t)\cos(\omega\Delta t+\varphi_0)]\,\mathrm{d}\varphi_0\mathrm{d}\Delta t. \tag{7}$$

We integrate over φ_0 and over p_y, to obtain the proper normalized 1D distribution

$$\rho(p_x) = \begin{cases}\frac{\mathcal{N}}{\pi\tau_e\bar{n}}\int\mathrm{d}\Delta t\frac{n(\Delta t)}{\Delta p_{\max}\eta(\Delta t)\sqrt{1-\frac{\Delta p_x^2}{\Delta p_{\max}^2\eta^2(\Delta t)}}}, & \Delta p_x^2 < \Delta p_{\max}^2\eta^2(\Delta t),\\ 0, & \Delta p_x^2 \geq \Delta p_{\max}^2\eta^2(\Delta t).\end{cases} \tag{8}$$

The change of the transverse momentum of the electron p_x is related to the distance x on the detector as $\Delta p_x = p_z x/L$, where L is the working distance and p_z is the z momentum component. The integrand is shown in Figure S2.a.

In the infinitely long interaction limit $\tau_l \to \infty$ and $g = 1$, using the definition $\int n(\Delta t)\,\mathrm{d}\Delta t = \bar{n}\tau_e$ we obtain

$$\rho(p_x) = \frac{\mathcal{N}}{\pi\Delta p_{\max}\sqrt{1-\Delta p_x^2/\Delta p_{\max}^2}}, \tag{9}$$

which is a double-horn pattern produced on the detector. Next we assume Gaussian envelope for the effective interaction envelope $\eta(\Delta t) = e^{-8\ln 2\Delta t^2/2\tau_{\text{int}}^2}$, and a Gaussian envelope for the electron pulse $n(\Delta t) = e^{-8\ln 2\Delta t^2/2\tau_e^2}$, where $\tau_{\text{int}} \ll \tau_e$. The integral can be evaluated numerically, yielding a narrow peak function on a wide pedestal, see Figure S2.b.

We compute the variance $\mathrm{Var}_\rho(p_x) = \langle p_x^2\rangle_\rho - \langle p_x\rangle_\rho^2$ of the distribution

$$\mathrm{Var}_\rho(p_x) = \int_{-\Delta p_{\max}}^{\Delta p_{\max}} p_x^2\rho(p_x)\mathrm{d}p_x. \tag{10}$$

By definition p_x is proportional to $\Delta p_{\max}$, therefore from the scaling property of the distribution follows that $\sqrt{\mathrm{Var}_\rho(p_x)} \propto \Delta p_{\max}$. We verify this relation numerically (See Figure S3.d). We note that this result

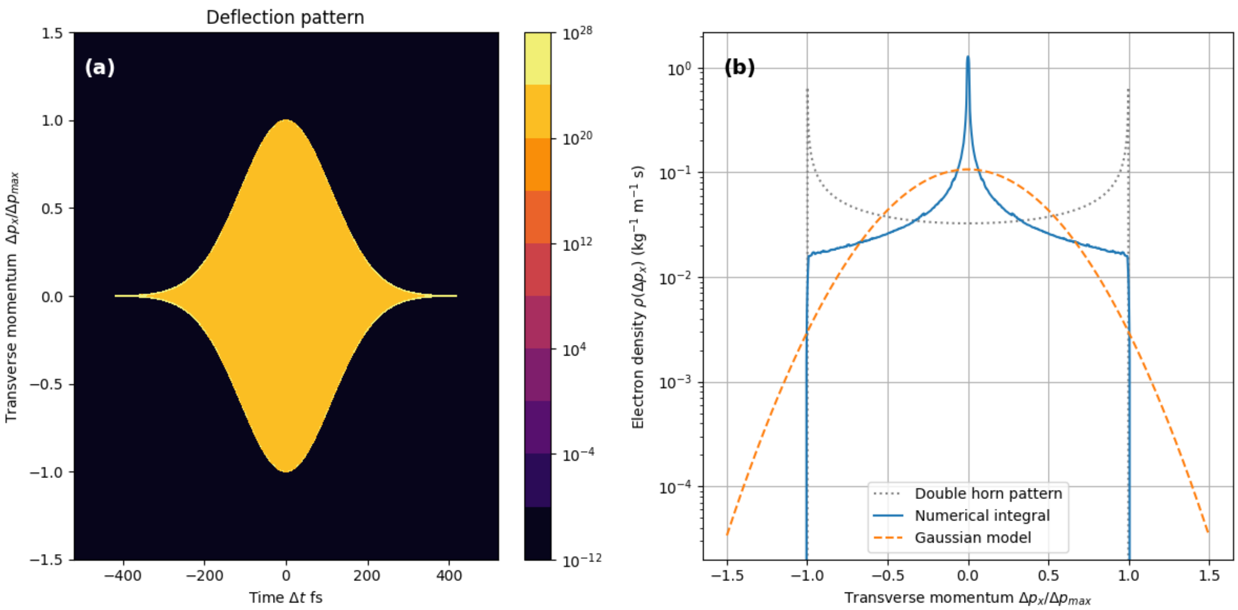

Figure S2: (a) Time dependent deflection pattern of the electrons in momentum space, (b) Electron density distribution: double-horn pattern for inifinitely long interaction (dot), numerically integrated model for 260 fs long interaction time and 800 fs long electron pulse (full) and approximation by a Gaussian model with equivalent variance (dash).

holds not only for the approximation where the effective interaction time of the electron is longer than the laser pulse duration $\tau_{\rm int} \gg \tau_l$, but also for the other limit case $\tau_{\rm int} \ll \tau_l$ or in the intermediate case $\tau_{\rm int} \approx \tau_l$. Additionally, the variance is independent off the relation of the electron pulse duration τ_e to τ_l and $\tau_{\rm int}$ or on the particular shape of either of the pulses.

For a clarifying model situation, we replace the 1D distribution $\rho(p_x)$ by a gaussian with the same variance $\sigma^2_{p_x 0} = {\rm Var}_\rho(p_x)$

$$f(p_x) = \frac{1}{\sqrt{2\pi}\sigma_{p_x}} \exp\{-p_x^2/2\sigma^2_{p_x 0}\}. \tag{11}$$

The comparison of the given distributions is shown in Figure S2.b.

Next, we include in our considerations the non-zero width of the electron beam. We still restrict the analysis to one dimension. The one dimensional distribution of the interacting electrons on the detector $D_{\rm 1D}(p_x, p_e, \sigma_{p_x 0})$ is given by the convolution between the profile of the non-interacting electron beam $h(p_x)$ with radius p_e and the distribution of deflected electrons for zero-width electron beam $f(p_x)$ with variance $\sigma_{p_x 0}$

$$\begin{aligned} D_{\rm 1D}(p_x, p_e, \sigma_{p_x,0}) &= \int_{-\infty}^{\infty} f(p_x - p'_x) h(p'_x)\, {\rm d}p'_x \\ &= \frac{1}{2}\left[{\rm erf}\left(\frac{p_x + p_e}{\sqrt{2}\sigma_{p_x 0}}\right) - {\rm erf}\left(\frac{p_x - p_e}{\sqrt{2}\sigma_{p_x 0}}\right)\right]. \end{aligned} \tag{12}$$

In realistic experimental conditions, the electrons primarily deflect along a dominant axis, which is given by the local orientation of the optical near-field. Here we assume that the electrons will deflect along $x-$axis in the 2D case.

The 2D distribution of the electron beam after interaction $D_{\rm 2D}(p_x, p_y, p_e, \sigma_{p_x 0})$ is the 2D convolution of the non-interacting beam $h(p_r)$ and the deflection pattern $\rho(p_x, p_y) \equiv \delta(p_y) f(p_x)$ defined by the gaussian

distribution along p_x and a delta function along p_y

$$D_{\rm 2D}(p_x, p_y, p_e, \sigma_{p_x0}) = \iint_{-\infty}^{\infty} h\Big(\sqrt{p_x'^2 + p_y'^2}\Big)\delta(p_y - p_y')f(p_x - p_x')\mathrm{d}p_x'\mathrm{d}p_y'. \tag{13}$$

The 2D distribution is normalized with respect to p_e such that:

$$\begin{aligned} &\iint_{-\infty}^{\infty} D_{\rm 2D}(p_x, p_y, p_e, \sigma_{p_x0})\,\mathrm{d}p_x\,\mathrm{d}p_y \\ &= \frac{1}{\pi p_e^2}\iint_{-\infty}^{\infty} D_{\rm 1D}(p_x, \sqrt{p_e^2 - p_y^2}, \sigma_{p_x0})h(p_y)\mathrm{d}p_x\,\mathrm{d}p_y = 1, \end{aligned} \tag{14}$$

where the parameter $\sqrt{p_e^2 - p_y^2}$ is the local spot width for a given p_y coordinate. Since $D_{\rm 2D}(p_x, p_y, p_e, \sigma_{p_x0})$ is an even function, the first moment in p_x is 0. The variance along p_x, averaged along p_y is then given by:

$$\begin{aligned} \sigma_{p_x}^2(p_e, \sigma_{p_x0}) &= \iint_{-\infty}^{\infty} p_x^2 D_{\rm 2D}(p_x, p_y, p_e, \sigma_{p_x0})\,\mathrm{d}p_x\,\mathrm{d}p_y \\ &= \frac{1}{\pi p_e^2}\int_{-\infty}^{\infty}\left(\frac{2}{3}\left(p_e^2 - p_y^2\right)^{\frac{3}{2}} + 2\sigma_{p_x0}^2\sqrt{p_e^2 - p_y^2}\right)h(p_y)\,\mathrm{d}p_y \\ &= \frac{1}{\pi}\int_0^{\pi}\left(\frac{2}{3}p_e^2\cos^4\varphi + 2\sigma_{p_x0}^2\cos^2\varphi\right)\mathrm{d}\varphi \\ &= \frac{1}{4}p_e^2 + \sigma_{p_x0}^2. \end{aligned} \tag{15}$$

In the second row, transform into radial coordinates was used $p_x = p_e\cos\varphi$, $p_y = p_e\sin\varphi$. The standard deviation of the distribution $\sigma_{p_x} = \sqrt{p_e^2/4 + \sigma_{p_x0}^2}$ scales linearly with the momentum distribution width and to the maximal transverse momentum transfer $\sigma_{p_x} \propto \sigma_{p_x0} \propto \Delta p_{\rm max}$. The contribution of σ_{p_x0} vanishes in regions far away from the nanostructure, or outside of the temporal overlap of the electron and the laser pulse and only the constant $p_e^2/4$ remains. It can be measured there and subtracted from the data.

An example of the electron beam spot with radius of 2.5 px before the deflection is shown in Figure S3.a. The deflection represented by a point spread function (8) is shown in Figure S3.b, and the deflection represented by a Gaussian model with equivalent variance is shown in Figure S3.c. We numerically calculated the variance for the spots for $\Delta p_{\rm max}$ in the range from 0 to 15 px and verify the analytical formula (15), for both models, also confirming that the distribution profile can be arbitrary, as long as the variance is the same, see Figure S3.d. The deviation from linear dependence on $\Delta p_{\rm max}$ is due to the finite pixel size and finite numerical time step in the integration of (8).

Numerical simulations of electromagnetic field distribution

The evolution of electric and magnetic fields in the vicinity of the periodic silicon nanostructure illuminated by a Gaussian pulsed beam with central frequency ω and linear polarization is calculated by numerically solving Maxwell's equations using Lumerical FDTD. We calculate the spatial distribution of the complex amplitudes of the near-fields $\tilde{\mathbf{E}}(\mathbf{r}, \omega)$, $\tilde{\mathbf{B}}(\mathbf{r}, \omega)$ generated at frequency ω using the Fourier transform of the time domain field. The excitation light is modeled as a plane wave incident perpendicular to the substrate of the nanostructure. Assuming the case of resonant interaction of electron and light the simulation area can be reduced to one period along the path of electrons. Further, based on the mirror symmetry of the structure with respect to x axis, a symmetric boundary condition can be used for polarization of light parallel to the trajectory of electrons, and antisymmetric boundary condition can be applied for polarization of light perpendicular to the trajectory of electrons. The simulation region has size of $26\,\mu\text{m} \times 7\,\mu\text{m} \times 0.62\,\mu\text{m}$ with symmetric/antisymmetric boundary condition on the x axis, perfect matching layer boundary conditions on the y axis, and periodic boundary conditions on the z axis. As a source, we used a plane wave. Adaptive mesh with the smallest step of 10 nm is used. The deflection corresponding to each electron trajectory is calculated in the classical approximation by using the transverse momentum change obtained from Eq. (1)

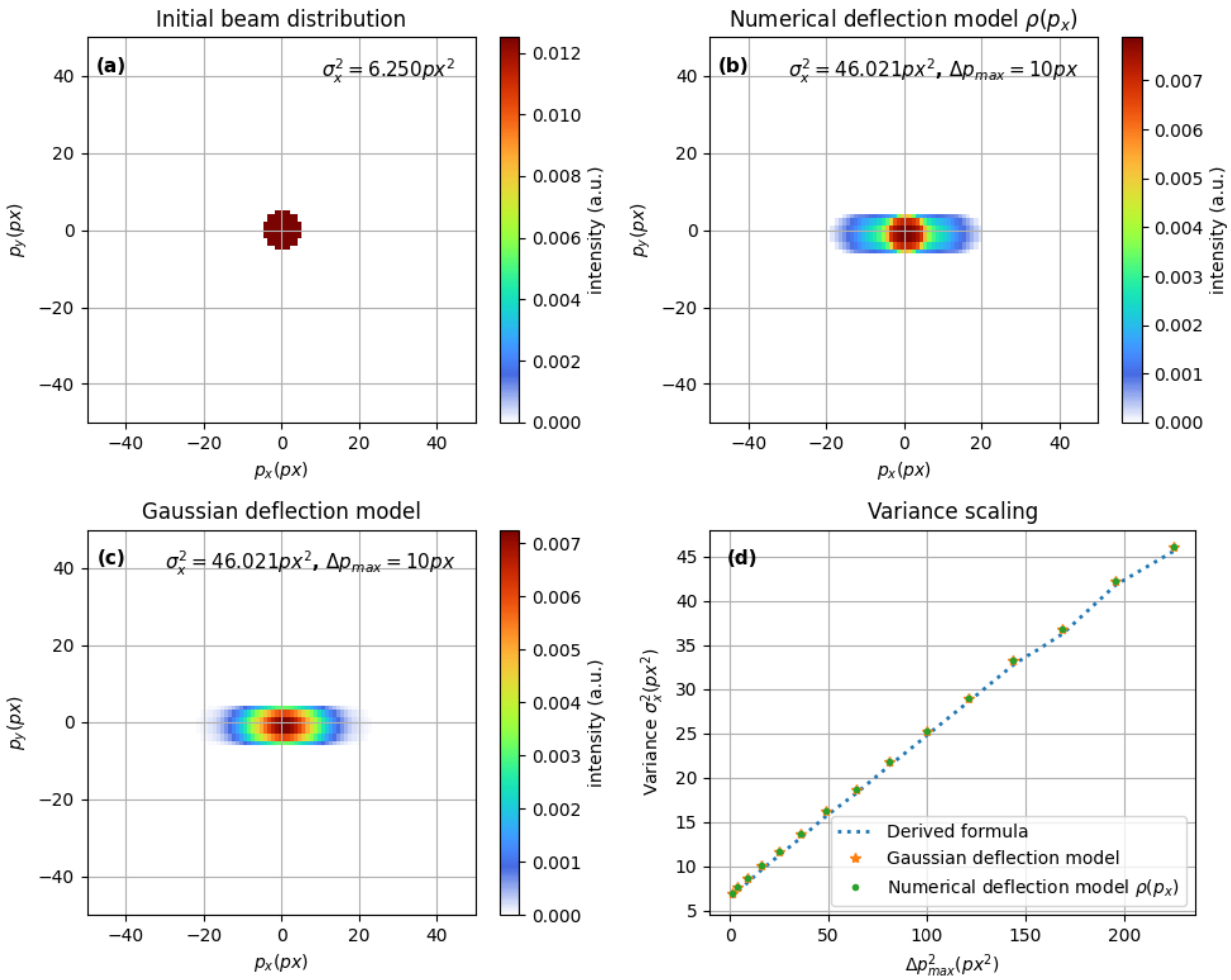

Figure S3: (a) Initial beam spot with radius of 2.5 detector pixels. (b) Beam spot with 1D numerically determined deflection kernel $\rho(p_x)$. (c) Beam spot with 1D Gaussian deflection kernel. (d) Beam spot variance as a function of $\Delta p_{\max}$ for the two models, compared with the analytical formula.

with the Lorentz force given by the time-domain fields $\mathbf{E}(\mathbf{r},t) = \Re\left\{\tilde{\mathbf{E}}(\mathbf{r},\omega)g(t-\Delta t)\exp(i\omega t + i\varphi_0)\right\}$ and $\mathbf{B}(\mathbf{r},t) = \Re\left\{\tilde{\mathbf{B}}(\mathbf{r},\omega)g(t-\Delta t)\exp(i\omega t + i\varphi_0)\right\}$.

Electrons are propagated through the calculated electromagnetic fields $\mathbf{E}(\mathbf{r},t)$ and $\mathbf{B}(\mathbf{r},t)$. This is repeated for every phase φ_0 and every point Δt in the sampling interval that covers the envelope function of the optical field $g(t-\Delta t)$. A normal distribution of electrons in time with FWHM duration of 800 fs is assumed. This creates a 2D electron momentum histogram at every scanned place of the sample, see equation (13) in Methods . From the 2D histogram standard deviations σ_{p_x} for x and σ_{p_y} for y coordinates are calculated. Since the ideal infinitesimally small electron beam spot size was assumed above ($P_e = 0$), the standard deviations are proportional to the cummulative deflecting force $\mathbf{F}_\perp$ (see Methods, Analytical formula for transverse momentum change). The standard deviations of the histogram scale linearly with the increasing number of simulated periods of the nanostructure.
